# The critical threshold of blood flow associated with spinal cord ischemia in a modified rabbit model developed by ligation of lumbar arteries

**DOI:** 10.1038/s41393-025-01071-3

**Published:** 2025-03-18

**Authors:** Li-Feng Xing, Ding-Wen Zheng, Yan-Song Miao, Yu-Cai Hong, Wei Xiao

**Affiliations:** 1 Department of Emergency Medicine, Sir Run Run Shaw Hospital, Zhejiang University School of Medicine, Hangzhou, China; 2 Department of Cardiac Surgery, Sir Run Run Shaw Hospital, Zhejiang University School of Medicine, Hangzhou, China

**Keywords:** Spinal cord diseases, Neurophysiology, Disease model, Spinal cord

## Abstract

**Study design:**

Animal study.

**Objectives:**

To investigate the influence of lumbar arteries ligation on spinal cord blood flow (SCBF), and to determine by what proportion the SCBF decrease would cause spinal cord ischemia (SCI) in rabbit model.

**Setting:**

Sir Run Run Shaw Hospital, Zhejiang University School of Medicine.

**Methods:**

SCI model was established by ligation of lumbar arteries in rabbits. 20 rabbits were divided into four groups: group A, sham surgery without ligation; group B, ligation at 3 levels; group C, ligation at 4 levels; group D, ligation at 5 levels. The SCBF was measured with laser doppler flowmetry, motor function was assessed using modified Tarlov grading system, and neurophysiological integrity was detected with motor-evoked potential (MEP), followed by histological observation on the seventh day after operation.

**Results:**

Lumbar arteries ligation at 3 levels led to average 40% decrease of SCBF, and spinal cord remained functional, electrophysiological and histological normal. Lumbar arteries ligation at 4 levels resulted in average 50% decrease of SCBF, slight motor dysfunction, prolonged latency of MEP and decreased cell volume of neuron, rabbits presented mild spinal cord injury. Lumbar arteries ligation at 5 levels caused average 60% decrease of SCBF, complete paraplegia, loss of MEP waveform and neuron karyopyknosis, rabbits presented severe SCI.

**Conclusion:**

More ligation of bilateral lumbar arteries leads to lower SCBF and increase the risk of SCI in rabbits, SCBF decreased by more than 50% could cause SCI. MEP associated significantly with SCBF, suggesting the usefulness of MEP to monitoring SCBF in surgery.

## Introduction

Despite intraoperative electrophysiological monitoring has been widely used to alert spinal cord injury, paraplegia caused by spinal cord ischemia (SCI) remains a devastating and unpredictable complication of operations in which interruption of aorta or segmental arteries is required, such as aortic repair surgery, and multilevel total *en-bloc* spondylectomy. Even though great improvements in surgery and intraoperative monitoring have been achieved, this complication still affects the quality of life severely. Unfortunately, lack of direct monitoring of spinal cord blood flow (SCBF) during surgery seems to be partly responsible for the occurrence of post-operative SCI. Though previous study suggested strong positive correlation between motor-evoked potential (MEP) and SCBF [1], and MEP can provide real-time feedback for strategic decision making [2], it is poorly defined that by what proportion the SCBF decreases will lead to abnormal MEP or SCI.

In our previous study, we established a modified animal model of SCI by selective ligation of the lumbar arteries in rabbits [3]. This model mimicked moderate and severe SCI by ligating different levels of lumbar arteries, more ligation of arteries led to more obvious change of MEP, and subsequently severer SCI [3]. Therefore, we assume that there may be a critical “threshold” of SCBF to maintain the normal function of spinal cord, when SCBF decrease to below this “threshold”, the occurrence of abnormal MEP and SCI may increase significantly. In this study, we aim to investigate the influence of bilateral lumbar arteries ligation on SCBF, and to explore that by what proportion the SCBF decrease can cause abnormal change of MEP and SCI in a modified rabbit model.

## Methods

### Study design

All experimental protocols of this study were approved by the Ethics Committee of Sir Run Run Shaw Hospital, Zhejiang University School of Medicine, and the study was conducted in accordance with the local legislation and institutional requirements. A total of 20 New Zealand rabbits (3-month age, weight 1.8–2.5 kg, average weight 2.2 kg) were used, in accordance with the 3 R rules and welfare for animal experimentation. The animals were randomly divided into four groups (n = 5): group A, underwent sham surgery without ligation of arteries; group B, ligation of bilateral lumbar arteries at 3 levels (L2-L4); group C, ligation of bilateral lumbar arteries at 4 levels (L2-L5); group D, ligation of bilateral lumbar arteries at 5 levels (L1-L5).

### Ligation of bilateral lumbar arteries

**Fig. 1:**
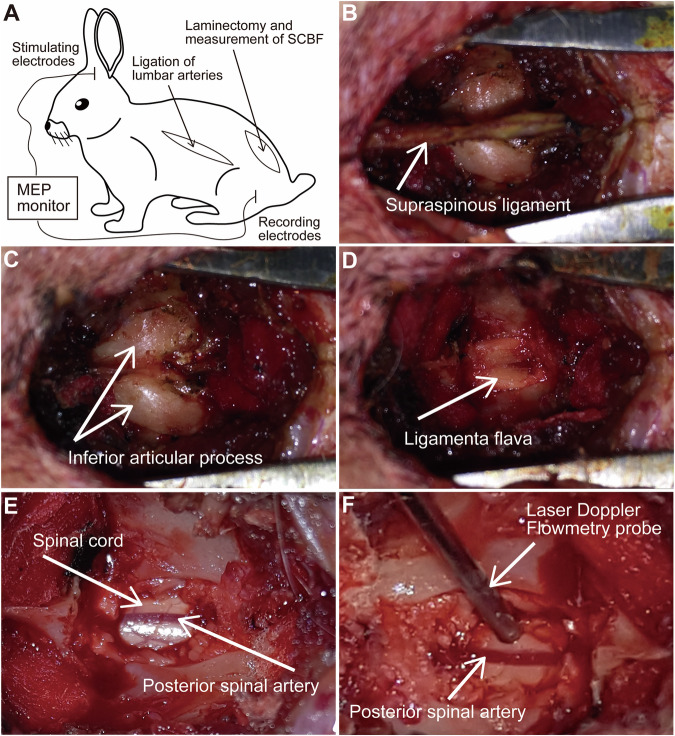
Diagram and operation of the methods in this study. **A** Diagram of MEP monitoring and surgical approach of laminectomy and lumbar arteries ligation. **B** Exposure of the dorsal side of the spine. **C** Exposure of bilateral inferior articular process. **D** Exposure of ligamenta flava after removal of inferior articular process. **E** Exposure of spinal cord and posterior spinal artery. **F** Measurement of SCBF using the laser doppler flowmetry probe.

The rabbits were fasted overnight for at least 8 h before operation, after anesthetized with pentobarbital sodium (35 mg/kg; Sigma, St Louis, MO, USA) by the ear vein injection, painful stimulation should not cause any pain reflex after anesthesia. Rabbits were then fastened on an operating table in right lateral decubitus position, with a heat lamp to maintain their body temperature at about 37°C. A left paramedian incision was made in the abdomen of rabbit (Fig. 1A), and the abdominal aorta and lumbar arteries were exposed through a retroperitoneal approach [3]. Bilateral lumbar arteries were ligated with silk and then cut off to make sure the segmental blood supply to spinal cord was interrupted completely. While rabbits in group A only underwent sham surgery, without artery ligation, group B had 3 pairs of lumbar arteries ligated, group C had 4 pairs of arteries ligated, and group D had 5 pairs of arteries ligated. Intramuscular injection of Penicillin was used intraoperatively and postoperatively to prevent wound infection.

### Electrophysiological test

The neurophysiological conductive function of spinal cord in rabbits was evaluated by motor evoked potentials produced by electrical stimulation (Cascade PRO with TCS-4 Transcranial Motor Stimulator and 16-channel PRO amplifier, Cadwell, Kennewick, WA, USA). Two stimulating needle-electrodes (Disposable Monopolar EMG Needle Electrodes, Cadwell, Kennewick, WA, USA) were placed on bilateral scalp at 1 cm cranial to the root of ears, and the compound muscle action potentials in bilateral anterior-thigh muscles was recorded with needle electrodes (Disposable Monopolar EMG Needle Electrodes, Cadwell, Kennewick, WA, USA). The stimulation signal used a rectangular waveform with the parameters: a pulse duration of 0.5 millisecond, an interstimulus interval of 2.0 millisecond, and a train of 4 pulses per stimulation at 1 Hz. The stimulation intensity, which ranged from 60 to 120 V, was set to 10% above the level that elicited the maximal amplitude according to the previous literature [4] and our experience obtained from lots of experimental practice [3]. The band pass filter was set at 10 to 3000 Hz. MEP were recorded after anesthesia (baseline), before ligation, 10 min and 1 h after ligation. At each timeline, MEP assessments were repeated at least 3 times to make sure the stability of MEP waveforms. The latency and amplitude of the first negative peak of MEP waves were measured, considering the amplitude of MEP might be more likely to be affected by anesthesia, we took the waveform obtained after anesthesia as baseline value. According to previous studies [5, 6], latency prolonged more than 10% or amplitude decreased more than 50% of baseline value was taken as a cut-off for abnormal MEP.

### Measurement of spinal cord blood flow

The vascular anatomy in New Zealand rabbit is similar to human, of which the segmental arteries supply both the blood flow of spinal cord and dura mater [7]. To measure the SCBF, a laminectomy was operated in each rabbit. The dorsal aspect of the spinal dura was exposed carefully, ensuring the anatomical intactness of spinal cord (Fig. 1B–E). The spinal cord blood flow was measured with Laser Doppler Flowmetry (LDF100C module, MP150 system; Biopac, Goleta, USA), the probe of Laser Doppler Flowmetry, 1 mm in diameter, was fixed to keep slight contact with the spinal dura without compression, and the contact point was about 1 mm lateral to the posterior spinal artery (Fig. 1F). Spinal cord blood flow was measured before ligation (baseline value), 10 min and 1 h after ligation. In each measurement, the signals collection using probes was continued for at least 5 s and then averaged as the value of SCBF, the unit of SCBF value was Blood Perfusion Unit (BPU). Data were given as mean ± standard deviation, and were analyzed by analysis of variance followed by a least significant difference test, difference with *P* values less than 0.05 was considered statistically significant.

### Neurological assessment

Postoperative motor function of lower limbs of rabbit was assessed at the 7th days after the surgery, according to the modified Tarlov grading system [8] and the Muscle function Grading system of International Standards for Neurological Classification of Spinal Cord Injury (ISNCSCI) scores [9]. For modified Tarlov grading system, grades were assigned as: 0, complete paraplegia with no hind extremity motion; 1, minor joint movements; 2, major joint movements; 3, the animals can stand; 4, the animals can walk; and 5, the animals can climb a 20° inclined plane. Data were given as mean ± standard deviation, and the analysis was performed with Kruskal-Wallis rank-sum test, difference with *P*-values less than 0.05 was considered statistically significant.

### Histopathological observation

After evaluation of neurological function on the 7th day, each animal was anesthetized with pentobarbital sodium (35 mg/kg) through the ear vein injection. The lumbar spinal column (from L1 to L5 vertebrae) together with paravertebral muscles were quickly cut off and placed in neutral buffered 10% formalin solution, and the animal was sacrificed using an overdose injection of pentobarbital sodium. After fixed in formalin solution for 5 days, the lumbar spinal cord was dissected out and placed in different concentration of alcoholic solutions for dehydration. The spinal cords were cut out at L3/4 level and then embedded in paraffin. Sections were cut at 5μm and were stained with hematoxylin–eosin (H&E) and Nissl’s staining. Cell counts of normal and damaged neurons of different groups were calculated and analyzed with *t*-test. The micro-structures in anterior horn of spinal cord ware observed at 400× magnification.

## Results

### More ligations of lumbar arteries led to lower SCBF

**Fig. 2:**
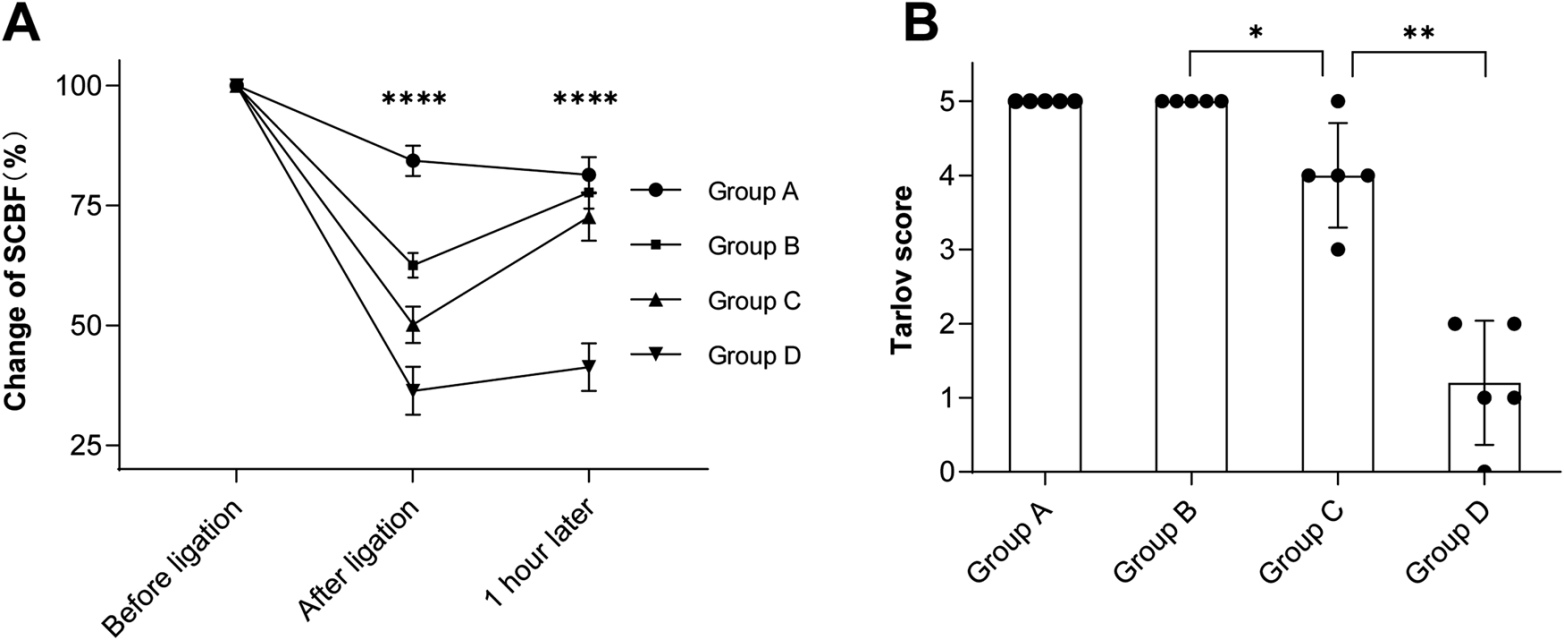
The change of SCBF and Tarlov score of rabbits. **A** Change of SCBF by each timepoint, before lumbar arteries ligation, 10 min after ligation and 1 h after ligation, respectively. **B** Tarlov score of each group. Data was presented as mean ± standard deviation, * *P* < 0.05, ** *P* < 0.01, **** *P* < 0.0001.

The measured values of SCBF of four groups by time were showed in Fig. 2A. There was no significant difference in the baseline values among groups. However, as more lumbar arteries were ligated, the SCBF decreased more significantly. SCBF decreased to average 84.3 ± 3.2% of baseline value in group A, 62.6 ± 2.6% in group B, 50.1 ± 3.8% in group C, and 36.4 ± 5.0% in group D at 10 min after ligation. Interestingly, rabbits had their SCBF recovered at 1 h after ligation. However, as more arteries had been ligated, less recovery was observed. SCBF recovered to average 81.38 ± 3.7% of baseline value in group A, 77.7 ± 4.3%in group B, 72.66 ± 5.0% in group C, and 41.28 ± 6.2% in group D. These results suggested that ligation of ≤4 pairs of lumbar arteries caused transient and reversible decrease of SCBF. As for rabbits in group D, ligation of 5 pairs of lumbar arteries led to persistent and irreversible decrease of SCBF.

### SCBF reduced by more than 50% increased the risk of SCI

**Fig. 3:**
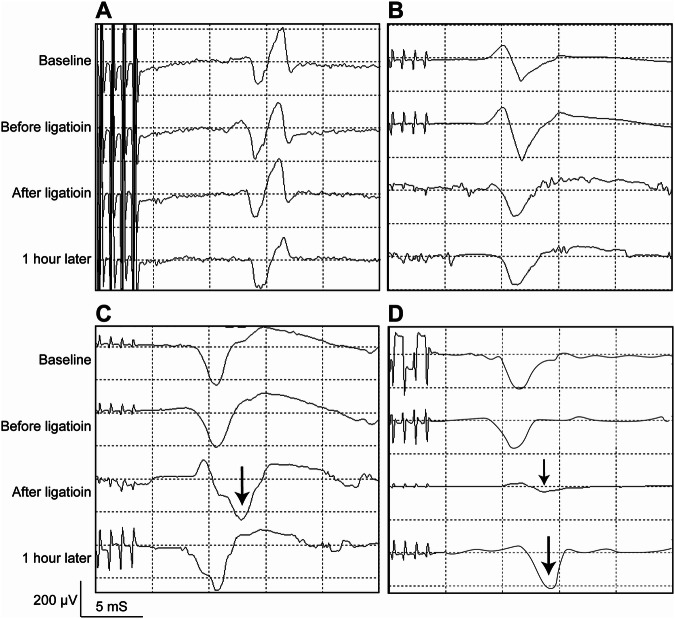
Representative change of MEP waveforms in each group. **A** Latency remained normal after ligation in group A. **B** Latency remained normal after ligation in group B. **C** Latency prolonged transiently after ligation in group C. **D** Amplitude decreased and latency prolonged after ligation in group D. The arrows indicated the prolonged latency of the first negative peak.

**Fig. 4:**
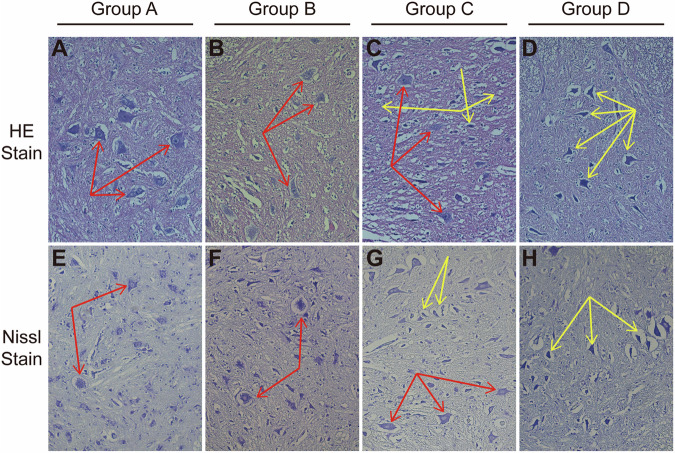
HE (A-D) and Nissl’s (E-H) staining of spinal cord sections after operation. **A, B, E, F** No sign of neuronal damage was observed in Group A and Group B. **C, G** Decrease of the neurons cell volume were observed in Group C. **D, H** Decrease of the neuron cell volume and karyopyknosis were observed in Group D. The red arrows indicate for normal neurons, and yellow arrows indicate for decreased cell volume or karyopyknosis of neurons.

The specific values of MEP latency and amplitude of each group at each time point are listed in Supplementary Table 1 and Supplementary Table 2 respectively. As mentioned above, the average SCBF stayed higher than 60% of baseline value in group A and B after arteries ligation (Fig. 2A). Accordingly, rabbits in these two groups graded 5 of Tarlov score and Muscle Function Grading (Fig. 2B), and had no significant change of MEP during experiment (Fig. 3A, B), and had no abnormal histological change in spinal cord (Fig. 4A, B, E, F). These results suggested that maintaining SCBF above 60% of baseline is safe for spinal cord in rabbits.

In group C, the average SCBF decreased by 50% of baseline value (Fig. 2A). Meanwhile, their Tarlov score and Muscle Function Grading ranged between grade 3 to grade 5 (4.0 ± 0.7), four rabbits presented moderate spinal cord motor dysfunction in this group (Fig. 2B), and prolonged of MEP latency was observed (Fig. 3C), and decreased cell volume of neuron was observed in the anterior horns of spinal cord by histological observation (Fig. 4C, G). These results suggested that SCBF decrease by 50% is risky of SCI in rabbits.

**Fig. 5:**
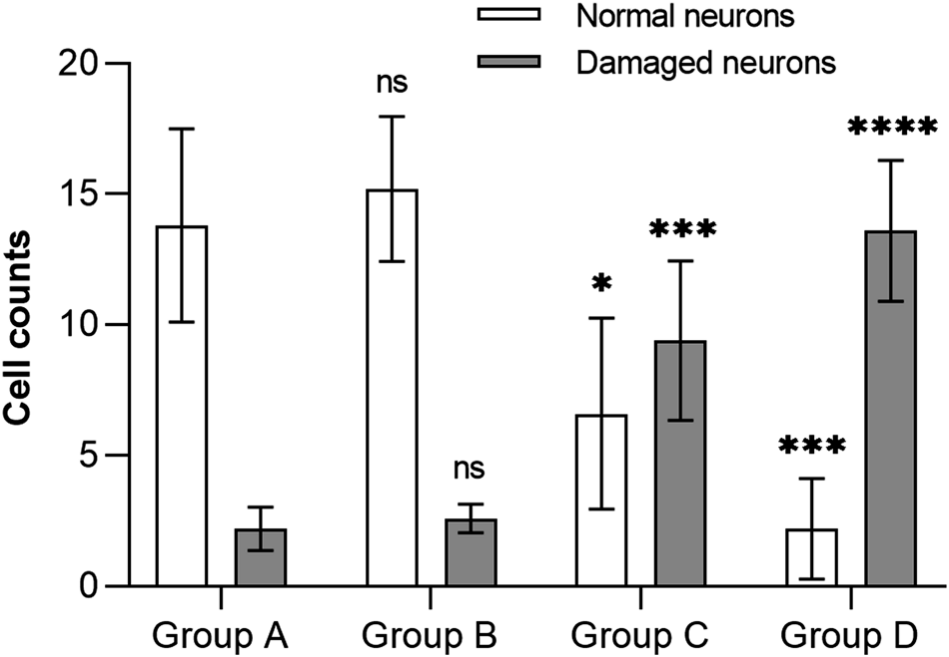
Cell counts of normal and damaged neurons on histologic section of different groups. Data was presented as mean ± standard deviation, * *P* < 0.05, *** *P* < 0.001, ****, *P* < 0.0001.

In group D, the average SCBF decreased by more than 60% of baseline value (Fig. 2A), Accordingly, all rabbits suffered severe motor dysfunctions, their Tarlov scores and Muscle Function Grading ranged between grade 0 to grade 2 (1.2 ± 0.8), one rabbits presented complete paraplegia (Fig. 2B), significant latency prolongation and amplitude decrease of MEP waveforms was recorded in this group (Fig. 3D), and neuron karyopyknosis was observed in the anterior horns of spinal cord by histological observation (Fig. 4D, H). Cell counts of normal and damaged neurons on histologic section of different groups were shown in Fig. 5, as more arteries ligated in Group C and D, less neurons survived. These results suggested that SCBF decrease by 60% would inevitably cause SCI in rabbits.

## Discussion

Previous researches demonstrated that the spinal cord vascular system in rabbit is similar to that in human [7]. In addition to anterior and posterior spinal arteries, spinal cord is nourished by segmental arteries which originate from aorta and give off radicular artery, radicular artery further emits a smaller branch named radiculomedullary artery to nourish the spinal cord and spinal dura [10]. Particularly, there is a very important radiculomedullary artery named Adamkiewicz artery feeding the thoracolumbar spinal cord [11]. Therefore, ligating segmental arteries reduces the SCBF and then increases the risk of SCI. SCI is always of great interest in the field of neurology and spinal surgery, to date, many animal models of SCI have been developed, and most of them were achieved by clamping the aorta transiently or constantly [12, 13], but these models are unable to induce consecutive decrease of SCBF. In our previous study [3], SCI model was established by ligation of lumbar arteries in rabbit, in which the Adamkiewicz artery mostly originates from L6 lumbar artery [7]. In this study, we further investigated the change of SCBF in rabbits by selective ligation of consecutive lumbar arteries from L1 to L5 level, avoiding interrupting the Adamkiewicz artery. Compare to group B in which lumbar arteries were ligated at 3 levels and had no obvious spinal cord dysfunction, group C and group D underwent extra L1 and L5 lumbar artery ligation, in which SCBF decreased by more than 50% and rabbits presented mild and severe SCI respectively. However, abnormal histological changes were observed at L3/4 level of spinal cord, suggesting that the SCI in these two groups could neither be attributed to single ligation of L1 nor L5 lumbar artery. These results demonstrated that SCBF decreased by more than 50% would be markedly risky of SCI when ligating ≥4 pairs of lumbar arteries in rabbit.

Fujimaki and Ueda et al. found that ligation of segmental arteries at ≤4 levels in dogs was safe but the SCBF was reduced to 70% of the normal value [14]; when ligation of segmental arteries was at ≥5 levels, SCI was induced and the SCBF was reduced to 40% to 50% of the normal value [15]. And Kato et al. concluded that interruption of bilateral segmental arteries at ≥4 consecutive levels reduce SCBF by 50% and risk producing ischemic spinal cord dysfunction [16]. Interestingly, our research using rabbit models demonstrated similar findings. In our study, ligation of lumbar arteries at ≥4 pairs in rabbits could induce SCI, and the SCBF decrease by more than 50% of the baseline. These findings pointed out that, though the level of segmental arteries need to be ligated to induce SCI varies in different animal species, it seems there is a common “threshold” of blood flow to maintain the normal function of spinal cord. According the findings mentioned above, we suggest that 50% of baseline SCBF value seems to be the critical “threshold”, SCBF decrease to lower than 50% are likely to induce SCI.

Electrophysiological techniques have been widely used for intraoperative neural monitoring to avoid spinal cord injury [5], whether electrophysiological monitoring indicate the change of SCBF remains unclear. As no specific and direct method to monitor SCBF has been developed yet, investigating the correlation between electrophysiological waveform and SCBF will help to make the best of electrophysiological monitoring. Previous clinical study also found intraoperative electrophysiological monitoring being important and useful measures to predict and prevent spinal cord ischemia in aortic repair surgery [17]. However, it has not been illustrated that how much the SCBF decrease will causes abnormal change of MEP. In this study, we found that ligating ≥4 pairs of lumbar arteries led to more than 50% decrease of SCBF, and caused prolonged latency or loss of MEP as well.

Interestingly, the SCBF increased significantly one hour later after artery ligation, except Group D, suggesting that mild decrease of SCBF caused by de ligation of lumbar arteries could be compensated by the collateral vascular network of spinal cord. Extensive anatomical researches on animal and human cadavers had shown an extensive vascular network within and surrounding the spine and paraspinal structures [18, 19], and growing evidence suggests that SCI is correlated more with the number of segmental arteries sacrificed rather than the sacrifice of a single important artery such as the Adamkiewicz Artery [20], therefore, recent studies have been shining a spotlight on the importance role of the collateral vascular network in preserving the spinal cord blood supply. This collateral network includes an extensive and interconnecting complex of vessels in the intraspinal and epidural spaces, paravertebral tissues, and paraspinous muscles [21].

To date, there is no method that can measure the SCBF directly in clinical practice, however, advances have been achieved in evaluating the SCBF indirectly by detecting the blood perfusion of collateral network. Near infrared spectroscopy (NIRS) offers non-invasive monitoring of tissue oxygenation, the most common application of NIRS is the assessment of cerebral oxygenation using stickers placed on the patient’s forehead [22]. According to the “collateral network” concept, both the paraspinous vasculature and spinal cord are fed by the segmental arteries, a theory was proposed that blood perfusion in the paraspinous vasculature may directly correlate with the blood perfusion in spinal cord [23], and the regional oxygenation in paraspinous muscle reflects the oxygenation in spinal cord tissue [24]. Previous clinical and animal studies have shown that paraspinous muscles NIRS may be a valuable noninvasive tool for monitoring spinal cord ischemia, since it reacts to segmental arteries occlusion and aortic cross-clamping, and correlates with spinal cord perfusion and neurologic outcome [25, 26].

Another example of take advantage of the collateral network is selective segmental artery endovascular coil embolization to prime the paraspinous collateral network before thoracoabdominal aortic aneurysm repair surgery [27, 28]. After segmental artery embolization, ischemic arteriogenic preconditioning of the collateral network was triggered, thereby allowing for recruitment of redundant arterial collaterals to the spinal cord [29]. This technique has been proven to be an effective tool for reducing the incidence of SCI in various experimental settings and numerous clinical pilot studies [30]. More valuable clinical applications of paraspinous collateral network remain to be discovered.

However, there are several limitations we should acknowledge in this study. Firstly, since only the dorsal part of spinal cord could be exposed by surgery, and then the probe of Laser Doppler Flowmetry was just placed on the surface of spinal dura, therefore, we only measured the SCBF that surrounding posterior spinal artery, and the SCBF that surrounding anterior spinal artery was not evaluated, which may not exactly reveal the overall change of blood flow in spinal cord. Secondly, we only focused on the tissue change of L3/4 spinal cord segment, which was already exposed and easily obtained, however, the entire spinal cord was not extracted for a more comprehensive histological observation, therefore a fully capture of the tissue change of spinal cord was not provided by this study. Thirdly, the results from animal model cannot be easily extrapolated to humans as the anatomical difference, but these findings are consistent with previous knowledge, which may contribute to better understand of SCI and provide useful tips to evaluate the utility of MEP in clinical intraoperative monitoring. Fourthly, anesthesia may have some effects on MEP results, though inhalational anesthetics or muscle relaxants were not used in our experiments, and we take the post-anesthesia MEP waveform as baseline value, the potential impact of anesthesia on MEP results was not totally addressed.

## Conclusion

More ligation of lumbar arteries leads to lower SCBF in rabbits. less than 50% decrease of baseline value seems to be the safe “threshold” of SCBF. SCI and abnormal change of MEP may by induced when SCBF decreased by more than 50%. And MEP waveforms correlated well with both SCBF and spinal cord motor function. Intraoperative MEP monitoring could indicate significant reduction of SCBF and predict postoperative SCI in this rabbit model, presenting the potential to be an alarm indicator to evaluate real-time SCBF and help to prevent postoperative SCI in clinical practice.

## Supplementary information

Supplementary Table 1.

Supplementary Table 2.

## Data Availability

The specific values of MEP latency and amplitude of each group at each time point are listed in Supplementary Table 1 and Supplementary Table 2 respectively.
